# Differences in Speech Recognition Between Children with Attention Deficits and Typically Developed Children Disappear When Exposed to 65 dB of Auditory Noise

**DOI:** 10.3389/fpsyg.2016.00034

**Published:** 2016-01-29

**Authors:** Göran B. W. Söderlund, Elisabeth Nilsson Jobs

**Affiliations:** ^1^Department of Teacher Education and Sports, Sogn og Fjordane University CollegeSogndal, Norway; ^2^Department of Psychology, Karolinska University HospitalStockholm, Sweden

**Keywords:** speech recognition, ADHD, Hagerman test, speech in noise, white noise, stochastic resonance

## Abstract

The most common neuropsychiatric condition in the in children is attention deficit hyperactivity disorder (ADHD), affecting ∼6–9% of the population. ADHD is distinguished by inattention and hyperactive, impulsive behaviors as well as poor performance in various cognitive tasks often leading to failures at school. Sensory and perceptual dysfunctions have also been noticed. Prior research has mainly focused on limitations in executive functioning where differences are often explained by deficits in pre-frontal cortex activation. Less notice has been given to sensory perception and subcortical functioning in ADHD. Recent research has shown that children with ADHD diagnosis have a deviant auditory brain stem response compared to healthy controls. The aim of the present study was to investigate if the speech recognition threshold differs between attentive and children with ADHD symptoms in two environmental sound conditions, with and without external noise. Previous research has namely shown that children with attention deficits can benefit from white noise exposure during cognitive tasks and here we investigate if noise benefit is present during an auditory perceptual task. For this purpose we used a modified Hagerman’s speech recognition test where children with and without attention deficits performed a binaural speech recognition task to assess the speech recognition threshold in no noise and noise conditions (65 dB). Results showed that the inattentive group displayed a higher speech recognition threshold than typically developed children and that the difference in speech recognition threshold disappeared when exposed to noise at supra threshold level. From this we conclude that inattention can partly be explained by sensory perceptual limitations that can possibly be ameliorated through noise exposure.

## Introduction

Attention deficit hyperactivity disorder (ADHD) is the most common neuropsychiatric condition in children, affecting ∼6–9% of the youth population and 3–5% of adults (e.g., Froehlich et al., 2007; Dopheide and Pliszka, 2009). ADHD is more prevalent among boys with a ratio of 1:3 (Biederman and Faraone, 2004; Lindemann et al., 2012), although these differences have diminished over the years and more girls are now diagnosed (de la Barra et al., 2013). The inattentive deficit comprises difficulties in sustaining attention, following instructions and being seemingly inattentive when spoken to directly, while the hyperactivity is manifested by overactivity, restlessness, and impulsivity (APA, 2013). Children with attention deficits display deficits in working memory, in particular auditory working memory (Alderson et al., 2015), often seem to have a listening problem, need auditory information to be repeated, have difficulties in dichotic listening tasks (Cacace and McFarland, 2006) and often display a sluggish cognitive tempo (McBurnett et al., 2001). ADHD is commonly associated with school failures and academic under-achievement (Faraone et al., 1993; Barkley et al., 2006; Serra-Pinheiro et al., 2008). A common explanation to symptoms of ADHD is low continuous levels of dopamine in the synaptic cleft (Volkow et al., 2009). In line with this, stimulant medication, e.g., methylphenidate, can be used to treat symptoms of ADHD, both behavioral and cognitive problems, to facilitate adaptation to school demands (Evans et al., 2001; Greenhill et al., 2002; Scheffler et al., 2009; Wigal et al., 2011). However, the best dose for optimal cognitive functioning has been found to be lower than the best dose for school behavior (Hale et al., 2011). Of greater concern, it is not evident that stimulant medication improves learning processes (Hellwig-Brida et al., 2011; Ginsberg and Lindefors, 2012), long term effects of medication are not well-known yet (Group, 2004) and neither are the effects on the developing brain (Anderson et al., 2002; Andersen, 2005). These uncertainties about medication make it urgent to look for alternative ways of improving attention and thus school performance for children with attention deficits.

The aim of the present study is to investigate if performance in speech recognition thresholds differs between children with ADHD symptoms and typically developed children (TDC) performing a speech recognition task in two different noise conditions, no noise and in 65 dB slightly modulated noise (that resembles white noise). The hypothesized difference between groups in speech recognition thresholds will here be further investigated. A reason for this is that prior research on ADHD has mainly focused on executive functioning where differences in performance are explained by deficits in pre-frontal cortex activation (e.g., Aaron et al., 2004; Brennan and Arnsten, 2008; Boonstra et al., 2010). Less notice has been given to sensory perception and subcortical functioning in ADHD even though there is a large overlap between central auditory processing disorder and ADHD (Riccio et al., 1994; Chermak et al., 2002).

There are somewhat contradictory findings regarding auditory perception in ADHD, indicating impairments and as well as no impairments. Some studies indicate differences between ADHD and TDC in speech processing, e.g., ADD children seems to prefer lower loudness levels when listening to speech, and display inferior speech discriminating ability when exposed to noise (Geffner et al., 1996; Lucker et al., 1996) and in hearing ability (Abdo et al., 2010). In binaural speech recognition tasks younger children with ADHD perform worse than TD children but at the same level in signal detection tasks (Pillsbury et al., 1995). In dichotic listening tasks TD children outperform children with ADHD in cognitive control of auditory input (Dramsdahl et al., 2011; Oie et al., 2014). From this we can conclude that ADHD children display a reduced signal recognition or perception efficiency but not for signal detection *per se*. Noise can be detrimental for attention but when investigating efferent auditory system the ability to suppress contralateral noise between an ADHD- and a control group was reported as equal (Pereira et al., 2012). Differences in auditory brainstem responses are found in ADHD and ASD patients that might indicate a fundamental difference in auditory processing compared to TDC (Källstrand et al., 2010; Claesdotter-Hybbinette et al., 2015; Jafari et al., 2015). To sum up, mixed results referred above provide good reasons to further investigate the topic of auditory perception and in particular speech recognition in ADHD in different noisy environments that are common during schoolwork.

The effects of acoustic noise on learning have often been investigated in relation to hearing in difficult conditions, where noise is usually an obstacle (Ljung et al., 2009; Song et al., 2012). Even low levels of continuous or intermittent noise are found to impair the learning and reproduction of texts in healthy control subjects (Trimmel et al., 2012). In contrast to the main body of evidence there have been an increasing number of studies reporting findings that loud acoustic random noise (80 dBA) under certain circumstances can be beneficial for performance on various cognitive tasks. This noise benefit is found in particular in individuals with an ADHD diagnosis (Söderlund et al., 2007) or with poor attention ability (Söderlund et al., 2010; Helps et al., 2014). Road traffic- and speech noise can also be beneficial for cognitive performance (Stansfeld et al., 2005; Söderlund and Sikström, 2012). This is a somewhat counter intuitive finding, while persons with attention problems are often shown as particularly vulnerable to distraction (e.g., Geffner et al., 1996; Rickman, 2001). A recent theory of noise benefit is the moderate brain arousal model (MBA) that relies on the phenomenon of stochastic resonance (SR; Sikström and Söderlund, 2007). SR is a ubiquitous phenomenon that exists in nature in any system with noise and a signal that requires passing a threshold as in the nervous system (McDonnell and Abbott, 2009). The simplest form of SR is threshold SR when a weak auditory signal is presented below the hearing threshold and becomes detectable when a random noise is added to the signal pushing it over the detection threshold (Stacey and Durand, 2001; Moss et al., 2004). In threshold SR the signal should be presented just below the hearing threshold and the noise in the same range (20–35 dB) for SR to occur. In supra threshold SR (SSR) this will occur when all noises added equals the mean of the signal amplitude (Stocks, 2000; McDonnell et al., 2007). This means that both noise and signal can be far above the hearing threshold; in the present study we focus on supra threshold SR setting the noise level at a constant level of 65 dB SPL and modulating the speech signal from 85 dB SPL and downward. The SR effect appears highly sensitive to both the intensity of the signal and the noise level; this relationship follows an inverted U-curve function, where performance peaks at moderate noise levels. This means that a moderate level of white noise is beneficial for performance whereas too little does not add the power required to bring the signal over the threshold and too much overpowers the signal, leading to a deterioration in attention and performance (McDonnell and Ward, 2011). The novel aspect of the MBA model is that it proposes individual differences in the SR effect and that these differences are linked to attention ability, while inattentive- or ADHD diagnosed individuals need higher input of noise compared to TDC to function at their full potential (Sikström and Söderlund, 2007).

In accordance with the MBA model this leads to the prediction that children with ADHD will benefit more from noise than children with normal attention, for whom noise will have a detrimental effect on performance. Accordingly, we will investigate if thresholds in speech recognition differ between children with ADHD symptoms and a typically developed control group and study how noise exposure affects the two groups. The hypothesis is that the noise during a speech recognition task will strengthen the signal and thus increase the signal-to-noise ratio in particular for the ADHD group; this improvement will be mediated through the supra threshold SR phenomenon. Our more specific predictions are as follows: (i) in the no noise condition the inattentive group demand a higher speech signal level as compared to controls in order to perceive the speech signal correctly due to a smaller signal-to-noise ratio; (ii) in the noisy condition (65 dB SPL modulated noise) these differences will disappear while noise strengthens the speech signal for the inattentive group and they will perform in parity with the TDC group.

## Materials and Methods

### Participants and Recruitment

Forty-nine secondary school boys between 9 and 10 years of age (*M* = 10,2) participated in the study. Girls were not included in the study since a vast majority of the clinical group were boys and gender could therefore be a confounding variable. Initial testing and parent- and teacher ratings of ADHD-symptoms were performed before the speech recognition task. In the ADHD group 10 boys were recruited by the staff (nurses and psychologists) at neuro-psychiatric units within the pediatric healthcare in the Stockholm catchment area, all having a clinical diagnosis set by a pediatrician. One participant was excluded due to incomplete test data. The 39 participants recruited for the control group came from a school in a mixed demographic suburb of Stockholm. One participant was excluded due to incomplete test data and one due to low general ability (IQ < 80). According to the initial teacher and parent ratings of ADHD symptoms, six of the participants had significant ADHD-symptoms (a mean score below 2,5) and were moved from the control group to the ADHD symptom group. Thus in all 15 participants were included in the ADHD symptom group and the remaining 31 participants constituted the typically developed control group. Of note is that ADHD is a behavioral diagnosis, i.e., certain behaviors make up criteria for the diagnosis. To get a diagnosis the symptoms should not be explained by a general cognitive deficit and symptoms should be present in childhood (DSM-5, 2013). Diagnoses are mainly based on questionnaires where symptoms are rated (Martel et al., 2015). Symptoms of inattention and hyperactivity are often viewed as dimensional traits that exist to a greater or lesser degree in the population (Marcus and Barry, 2011). The ADHD symptom rating used in this study (see below) is based on the DSM-5 criteria and captures behaviors within the diagnostic realm. Note that the term *ADHD symptom group* is used when the extra six participants are included. Group assignments were made prior to the speech recognition test. All participation was followed after written permission from parents and oral consent from children. The regional ethic board in Stockholm approved the study.

The initial teacher- and parent ratings of participants covered items about school achievements (reading, arithmetic, oral presentation, general school performance), social skills, hearing and hearing sensitivity, language spoken at home, and medication. All participants had normal hearing according to self-report, parent and/or teacher reports. To rule out peripheral hearing loss, exclusion criterion was set to binaural hearing threshold of 37 dB SPL (equivalent to 15 dB HL) or below according to the result in the no-noise condition. No participants were excluded for this reason. ADHD symptom rating were based on the SWAN scales (Swanson et al., 2007), the TTI-IV interview manual (Tannock et al., 2002), and the Diagnostic and Statistical Manual of Mental Disorders criteria for ADHD (APA, 2000, 2013). The rating consisted of salutogenic items rated on a scale from 1 to 7 where 1 was much below average, 7 was much above average, 4 was average and 1 much below average. The rating covered nine questions about attention ability and nine about activity level. Two subtests, “Similarities” and “Picture concepts” from the Wechsler Intelligence scale for children, WISC-IV were used to measure cognitive ability. The “Similarities” subtest measures verbal fluid intelligence and the “Picture concept” subtest measures non-verbal fluid intelligence (Flanagan et al., 2006). Two subtests of auditory working memory were used, “Digit span” from Wechsler scale of Intelligence, WISC- IV and “Repetition of sentences” from the neuropsychological battery NEPSY (Korkman et al., 2001). The subtests “Score” and “Score- double task” (Score DT) from the TEA-Ch battery (Manly et al., 2001) were used to asses sustained auditory attention and auditory divided attention respectively. See participant characteristics in **Table 1.**

**Table 1:** Participant characteristics: cognitive test scores and ratings.

Cognitive ability and ratings	Control group Mean (SD)		ADHD-symptom group Mean (SD)	
Age in years	10,3 *N* = 31		10,1 *N* = 15	
*Cognitive abilities*			
Picture concepts, ss	10.4 (1.8)		9.1 (2.2)		
Similarities, ss	9.8 (2.4)		8.3 (2.5)^∗^		
*Working memory*			
Digit span forward ss	8.5 (2.2)		7.1 (2.0)^∗^		
Digit span backward ss	9.0 (2.5)		7.1 (1.9)^∗∗^		
Repetition of sentences ss	6.4 (2.1)		4.5 (1.2)^∗∗∗^		
*Sustained and divided attention*			
Score ss	8.2 (2.9)		6.2 (2.7)^∗^		
Score doubleT ss	9.2 (4.5)		4.9 (2.6)^∗∗∗^		

**Ratings**	**Parents *N* = 29**	**Teachers *N* = 31**	**Parents *N* = 9**	**Teachers *N* = 13**

Attention ability	4.5 (0.8)	4.4 (1.0)	2.6 (0.6)^∗∗∗^	2.5 (0.8)^∗∗∗^
Activity level	4.9 (0.9)	4.4 (1.0)	3.3 (0.7)^∗∗∗^	3.0 (1.0)^∗∗∗^
**Skills**				
Reading	5.0 (1.3)	4.4 (1.2)	3.9 (1.4)	3.4 (1.6)
Arithmetic	5.3 (1.1)	4.7 (1.2)	4.3 (1.5)	3.5 (1.2)^∗∗^
Oral communication	5.1 (1.3)	4.6 (1.0)	4.0 (1.4)	3.4 (1.2)^∗∗^
General school performance	5.1 (1.0)	4.6 (1.0)	3.3 (0.9)^∗∗∗^	3.3 (1.2)^∗∗^
Social ability with peers	5.3 (1.2)	4.6 (1.0)	4.7 (1.3)	3.5 (0.9)^∗∗∗^
Hearing	5.0 (1.2)	4.3 (0.7)	4.8 (1.0)	4.1 (0.6)


*Group comparison Welch’s F. ^∗^*p* < 0.05, ^∗∗^*p* < 0.01, ^∗∗∗^*p* < 0.001.*

*N = number of participants in each group and number of participants rated by parents and teachers.*

*ss = scaled scores 1–19. Rating scale: scores 1–7.*

### Materials and Test Battery

The signal-to- noise ratio (i.e., the relation between the signal and the noise in dB), where it is comfortable to listen to speech is about 15 dB, i.e., when the signal is 15 dB louder than the noise. Noise levels can thus be as high as 40–50 dB SPL without affecting speech intelligibility if the signal is presented at about 65 dB SPL. A comfortable level for listening to speech in quiet or low levels of background noise is about 60–65 dB SPL, which corresponds to the level of normal conversational speech heard at 1 m (Scharine et al., 2009). The ability to detect speech in quiet improves from the age of 4–10 years with 9 dB, i.e., one can hear speech on average 9 dB softer at the age of 10 years (Neumann et al., 2012).

Speech-in-noise tests, where the speech signal is imbedded in background noise, are mainly used for evaluation of the benefit of hearing aids but also for assessing auditory functioning in individuals that report difficulties in perceiving speech in noisy surroundings despite good peripheral hearing (i.e., tone thresholds). In the present study we used the Hagerman sentences test that is one of the Swedish speech recognition tests in noise used in clinical settings. The test has a fixed noise level where the speech signal is attenuated. The noise sounds like a continuous noise but is slightly modulated to resemble the temporal variations of speech (Hagerman, 1984, 2002). The slightly modulated noise resembles white noise but does not have a flat power spectrum, most of the energy is between 1 and 5 kHz, the frequencies of normal speech (Hagerman, 2002). Of note, slightly modulated noise possesses the same stochastic, random properties as white noise or pink noise. A children version of the Hagerman sentences test has been developed (Hagerman and Richardson, 2009) using three-word sentences, having the syntactic structure of numeral– adjective – object (e.g., “three beautiful gloves”). In this version, the slightly modulated noise is set to 65 dB SPL to be more comfortable for children and the threshold has been set to 68% correct words, i.e., two out of three words should be correctly repeated (Hagerman and Richardson, 2009). The ambition with present setting was to find out if there was a differential effect of noise on groups as such and not to specify effects at different levels. To use more than two noise levels would have given us more information but we choose to use the Hagerman version as close to the original test as possible. To develop and use a new non-validated test without norm data was not an alternative. Moreover, it would have prolonged the test considerably and put too much strain on the participants on cost of the reliability.

In the present study the test was presented binaurally with headphones. The equipment for the speech recognition test consisted of a lap-top, headphones Sennheiser HDA 280, and an external audiocard Behringer UCA 222 with the software calibrated at the department of Technical Audiology at Karolinska Institute in Stockholm. Thresholds in quiet (no noise) for the minimum audible level in dB SPL were tested in order to compare this condition with the noise condition at a well audible level. In addition, both speech and noise were presented simultaneously in both ears, in order to get a more natural hearing situation. The computer-based adaptive method adjusted the speech level after each sentence depending on how many words that was repeated correctly. In the first condition (noise), the sentences (i.e., signal) were presented at suprathreshold level at 85 dB SPL and then attenuated in slightly modulated noise at 65 dB SPL, in order to identify the threshold for the correct recognition, a criterion of 67% words, i.e., two out of three words correctly recalled. In the second condition (no- noise), the sentences were presented at 50 dB SPL and then attenuated until the minimum audible level using the same criterion as above. The test comprised in all 12 lists with 10 sentences in each list. From these 12 lists three randomized lists were chosen for each participant in each condition, one list for practicing and two for the actual test. Each participant was exposed to 30 sentences in each condition (i.e., three lists) in total 60 sentences. In some cases one, two and in the odd case three sentences occurred twice during a condition. The exposure to repeated sentences, were very similar to each group with slightly more repetitions in the ADHD-symptom group and in the no-noise condition. For the assessment of thresholds, the training list was used to make participants familiar with the task and the test situation and to set a suitable speech signal level to start the first test list from. The two test lists was built on a computer-based adaptive method that adjusted the speech level after each sentence depending on how many words the participant recognized correctly. If *no word* was recognized, the signal was increased 2 dB, if only *one word* was recognized the speech signal was increased by 1dB, if two words were recognized the signal remained at the same level and if all *three words* were recognized the signal was decreased with 1 dB. The speech level of the last sentence in the first test list decided the level at which to begin the second test list. The program calculated a mean of the 10 sentences for the last list.

### Design and Test Procedure

The design was a 2 × 2 mixed design. The within group manipulation was binaural speech recognition in two conditions no-noise vs. noise. Threshold in the no nose condition was set in dB SPL at minimum audible level (attenuated from 50 dB SPL). Speech recognition threshold in noise was set in dB SPL at well audible level (attenuated from 85 dB SPL) in noise at 65 dB SPL. The between group variable was children with ADHD symptoms vs. controls. Dependent variable was binaural speech recognition thresholds dB SPL in the no- noise condition vs. the noise condition.

#### Test Procedure

The testing was conducted at the child’ s school to minimize drop-out rate and participants were tested individually in a room during the school day by the same licensed psychologist and took part in the participants’ schools for optimal participation rate. The test session began with the modified Hagerman test for children. The two speech recognition conditions (no noise vs. noise) were given in counterbalanced order and took ∼10 min to perform in all. The test session also included tests for participant characteristics (**Table 1**). They were carried out in the same succession and administrated according to the manuals and took ∼40 min to administer. After 15 min of testing a short break with juice and fruit followed. After taking part in the testing, the boys received a movie voucher.

## Results

### Speech Recognition Thresholds

**FIGURE 1:**
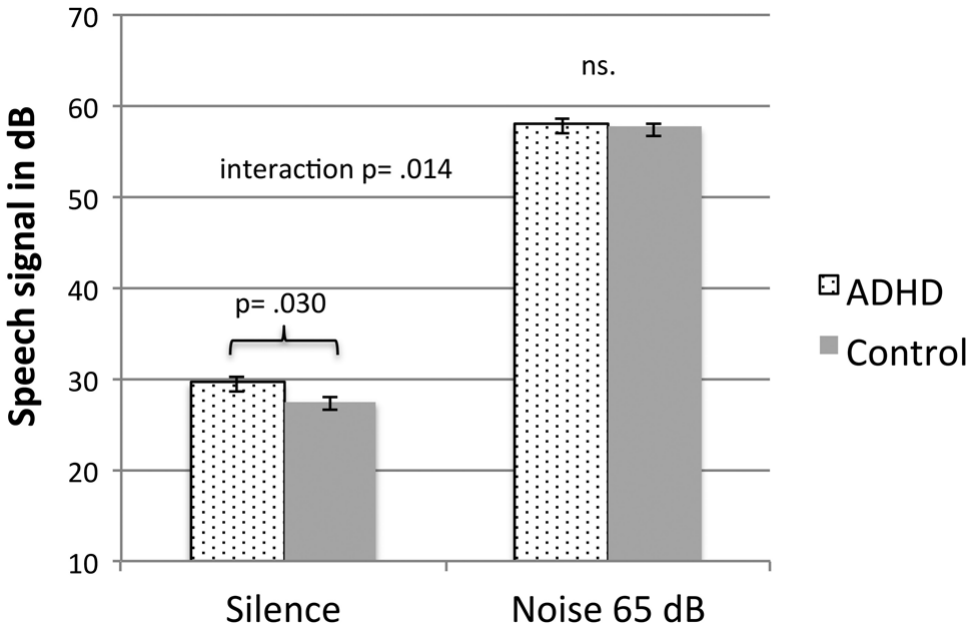
**Speech recognition thresholds for children with ADHD symptoms and TD children in a silence and in 65 dB modulated white noise.** Error bars represent standard error of the mean.

A 2 × 2 mixed analysis of variance (ANOVA) was conducted to asses speech recognition threshold with one between subjects factor, group (ADHD symptoms vs. TDC) and one within subjects factor, noise condition (no noise vs. 65 dB SPL modulated noise). A criterion of 67% words correct was used, two out of three words were correctly recalled for a correct response. We found a main effect of noise [*F*(1,44) = 6852.6, *p* < 0.0005] and an interaction between speech condition and group [*F*(1,44) = 6.52, *p* = 0.014, η^2^ = 0.129]. This means that 65 dB noise affected groups differently; in the no noise condition TDC children displayed a lower speech recognition threshold as compared to the ADHD symptom group. The overall differences between groups was significant [*F*(1,44) = 7.70, *p* = 0.008, η^2^ = 0.149]. In the noise condition both groups had similar recognition thresholds. *Post hoc* testing, an independent samples *t*-test showed that the difference between groups in the no noise condition was significant [*t*(44) = 2.36, *p* = 0.030] the TDC group perceived correctly at 27.6 dB while the ADHD symptom group needed 29.6 dB for correct performance. In the noisy condition this difference disappeared [57.7 vs. 58.0 dB; *t*(44) = 0.97, *p* = 0.336], see **Figure 1.**

We conducted an alternate mixed ANOVA that only included the originally clinically diagnosed ADHD group of nine children and the TD group of 31 control children. Data displayed that the interaction between groups increased further [*F*(1,38) = 11.79, *p* = 0.001, η^2^ = 0.237] and a *t*-test showed that the mean difference still was significant [*t*(38) = 3.32, *p* = 0.002]. ADHD children now require 30.4 dB for a correct recall and there was still no difference between groups in the noisy condition still [57.7 vs. 57.9 dB; *t*(38) = 0.40, *p* = 0.691]. Only one participant had a threshold just below the level for inclusion (i.e., 37 dB SPL/15 dB HL). However, if excluding this participant from the ADHD group, the difference between groups still remained significant [*t*(37) = 2.58, *p* = 0.014].

**FIGURE 2:**
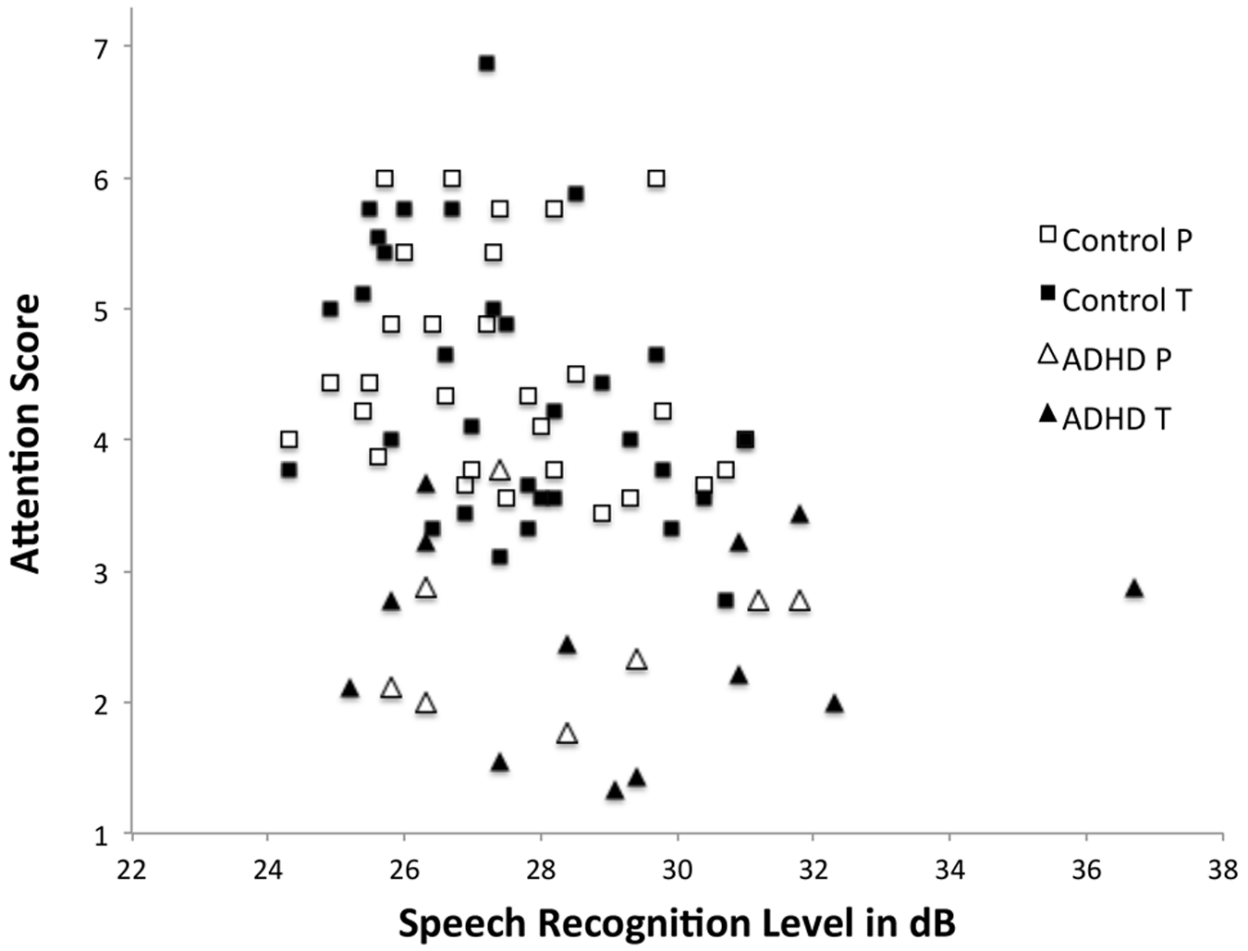
**Shows the relationship between speech recognition thresholds (in dB, y-axis) and scores of parent and teacher ratings of attention (x-axis: 1 = inattentive; 7 = high attention).** Filled symbols are teacher ratings and non-filled are parent ratings.

The relationship between speech recognition threshold in silence and attention ability was further investigated in a Pearson product-moment correlation, see **Figure 2.** Data showed a significant correlation between attention ability as rated by teachers (*r*^2^ = 0.385, *p* = 0.010) and parents (*r*^2^ = 0.342, *p* = 0.047). Hyperactivity by parent’s ratings and speech recognition was only significantly correlated (*r*^2^ = 0.437, *p* = 0.006), see **Table 2** for all figures. However, there were no further correlations between cognitive ability and speech recognition thresholds as measured by similarities (*r*^2^ = 0.007, *p* = 0.963) and picture completion (*r*^2^ = 0.102, *p* = 0.502).

**Table 2:** Bivariate correlation between speech recognition threshold, attention, and hyperactivity.

Measures	SRQ	ADDP	ADDT	HyP	HyT
Speech recognition in quiet (SRQ) *r*^2^	1				
*p*-value					
Attention parents (ADDP) *r*^2^	**0.324**	1			
*p*-value (*N* = 38)	0.047				
Attention teachers (AT) *r*^2^	**0.385**	**0.676**	1		
*p*-value (*N* = 44)	0.010	0.000			
Hyperactivity parents (HyT) *r*^2^	**0.437**	**0.779**	**0.676**	1	
*p*-value (*N* = 38)	0.006	0.000	0.000		
Hyperactivity teachers (HyT) *r*^2^	0.183	**0.549**	**0.747**	**0.552**	1
*p*-value (*N* = 44)	0.234	0.000	0.000	0.000	


*Significant values in bold.*

## Discussion

The current study tested the hypothesis that children who differ in attention (ADHD symptoms vs. TDC children) will have different speech recognition thresholds, which could be diminished in noisy conditions following the MBA model (Sikström and Söderlund, 2007). Firstly, the results corroborated the prediction that there was a difference between speech recognition thresholds, although the difference was small (just over 2 dB) and could possibly be within the margin of error, due to natural variation in sensitivity to sensory stimuli (Scharine et al., 2009). The correlation between hearing thresholds and the ratings of ADHD symptoms offers further arguments for the significance of the present finding. The results indicate that the difference is due to a real neurocognitive dimension rather than just a perceptual peripheral deficit or sensory fluctuation. The most important finding in the present study is the proposed link between attention ability and speech recognition. This is the first study, to the best of our knowledge, which has shown a link between perceptual speech thresholds and behavioral assessments of ADHD symptoms after parent- and teacher ratings. This means that different groups of individuals perceive auditory information differently and are furthermore differently affected by external noise. Of course this will need to be replicated in future studies. Secondly, and even more interesting, was that binaural noise exposure made these differences disappear; in the noisy condition both groups displayed almost exactly the same signal-to-noise ratio of ≈7 dB in order to achieve correct speech recognition at an audible level (≈58 dB).

The existence of group differences in auditory perception between ADHD and TDC has been reported earlier in a small number of studies. Pillsbury et al. (1995) found no deficits in signal detection *per se* in the ADHD group, but found reduced processing efficiency for signal recognition, in particular in noisy environments. This is of great interest for the present study while it provides arguments for distinguishing between signal detection and signal recognition in ADHD when discussing results on auditory perception. Present results could also indicate deficits in the auditory pathways in ADHD; however, Central Auditory Processing Deficit (CAPD) is a complex and heterogeneous group of auditory-specific disorders, usually associated with a range of listening- and learning deficits, including auditory discrimination. There is a huge overlap between language processing disorders and ADHD, in particular in the inattentive subtype (ADD; Chermak et al., 2002). A clinical study estimated the overlap between CAPD and ADD as high as 50% (Riccio et al., 1994). This finding is verified by Oie et al. (2014) who found impairments only in the predominantly inattentive group and not in the ADHD combined group in an executive auditory control task (dichotic listening). This goes along with data from the present study where inattention was found to be a stronger predictor of higher speech recognition thresholds than hyperactivity, as shown by the correlations in **Table 2.**

Working memory capacity can be a confounding variable when conducting auditory perception studies in ADHD samples. As shown in **Table 1**, the ADHD symptom group had a poorer working memory performance than the TDC group and this is hallmark of ADHD in general (e.g., Alloway, 2011; Kasper et al., 2012). ADHD-related working memory deficits reflect a combination of impaired central executive and phonological storage/rehearsal processes and performance deterioration when stimuli set sizes are increased (Alderson et al., 2015). This means that a phonological processing deficit could just mirror a limited working memory capacity and not phonological storage as such. For this reason many auditory processing tests might be invalid because of difficulties in dissociating auditory processing disorder from language-, attention problems, and working memory capacity (Katz and Tillery, 2005). With this in mind, the current study indicates that the Hagerman speech-in noise test for children is a robust test not loading on working memory. The difference in the no noise condition is thus likely to capture auditory processing differences rather than differences in working memory. At supra threshold level the difference between groups disappeared, despite inferior working memory performance in the inattentive children. From this it is tempting to draw the same conclusions about the threshold condition. There might be other processes that could mediate the auditory processes at this level that are not taken into account in the present study. However, the NEPSY sentence repetition test showed that the ADHD symptom group could repeat at least seven words while the Hagerman test only require three words to be repeated. The complicated interrelation between working memory capacity, attention, and speech recognition thresholds therefore needs to be further investigated. Research on auditory brain stem responses supports though the view that ADHD patients are affected on the auditory processing level rather than the cognitive, Two recent studies found that dysfunctions of the auditory brainstem pathways cause deficits in temporal encoding of both speech and non-speech stimuli that could explain speech-processing difficulties in ADHD (Claesdotter-Hybbinette et al., 2015; Jafari et al., 2015). Of note is that the study by Claesdotter-Hybbinette et al. (2015) is made on girls and the present study on boys, providing an argument for possible generalization of the present findings being valid for girls as well.

The effects of auditory noise can be both positive, e.g., lowering hearing thresholds (Zeng et al., 2000), and negative, but in fact mostly the latter, in particular in demanding cognitive tasks (Sörqvist, 2010). In the present study we focus on positive effects of noise referring to the effect of SR where noise under certain well-defined conditions can be beneficial for performance, in particular in nervous systems that are not working at their optimum (McDonnell and Ward, 2011). We found a noise benefit in ADHD and in line with this finding, Pereira et al. (2012) found that the ability to suppress contralateral noise in a ADHD- and a control group was equal. Further support was given by Behne et al. (2005), who showed that when exposing noise and signal into the same ear, in particular into the right auditory cortex, this would lead to greater brain activation, thus possibly making noise an advantage instead of an obstacle in processing complex auditory signals like speech. On the other hand, contradicting results was found by Abdo et al. (2010) were a group of normal hearing ADHD children performed worse than controls on both a digit dichotic listening task and on a speech-in-noise task. However, the kind of noise that was used during the task performance in this study is not described and of note is that the tasks in Abdo et al.’s (2010) study did put high demands on both auditory processing and working memory, not just auditory perception as in the present study. The type of noise also plays a pivotal role, e.g., if the noise is meaningless as in the present study (white noise like), or if it is meaningful, such as speech noise. For example, Hawley et al. (2004) found that binaural meaningless noise did not interfere with performance whereas meaningful (speech babble) monaural noise did. In a study by (Söderlund and Sikström, 2012) cafeteria noise, i.e., speech noise, was used and results showed exactly the same effect of cafeteria noise as the one of white noise, that is, a noise benefit for the inattentive group. Thus, there is a problem when comparing results form different studies when the type of noise sometimes is not properly described while this seems to play a pivotal role for the results.

Additionally, to yield a noise benefit it seems that the noise should be exposed binaural. In auditory perception tasks, different kinds of task-irrelevant noises are frequently used in experiments that can be presented both monaurally and binaurally. For example, in dichotic listening (DL, binaural) tasks it shows that ADHD patients have a reduced left hemisphere specialization, i.e., larger right hemisphere contribution, which leads to impaired word processing among ADHD patients when word processing is normally dedicated to the left hemisphere (Hale et al., 2006). Further support is given by Dramsdahl et al. (2011), where the ADHD patients failed to perceive syllables in the forced left ear condition in dichotic listening tasks, as the forced left condition is depending on activity in the right hemisphere. Of note is that ADHD and TDC performed equally well in the non-forced and forced right ear conditions linked to the ability to just perceive the syllables and not on top-down directed cognitive control. This provides further evidence that there is a distinction between the detection of a target and the perception or recognition of targets like word stimuli. Age and developmental factors can also play a role in speech perception, with younger children displaying a larger susceptibility to noise than older children (Talarico et al., 2007). From this we conclude that if noise benefit should occur in speech recognition tasks the noise has to be: binaural, meaningless, random, and within a moderate loudness range (65–80 dB) to provide opportunity for supra threshold SR to occur (McDonnell et al., 2007). Evidence of the need to take contextual factors into account is provided from Michalek et al. (2014) posing that factors such as diagnosis, modality, and signal-to-noise ratio all have a main effect on a person’s ability to process speech in noise. To sum up, auditory processing of speech is influenced by both internal (e.g., attention, age, working memory, brain stem response) and external factors (e.g., noise type, bi- or monaural, visual information).

Stimulant medication in ADHD seems to have a robust effect on ADHD behaviors (Antshel et al., 2014) and on cognitive task performance (Murray et al., 2011), but not as obvious when it comes to school performance (Hellwig-Brida et al., 2011; Wigal et al., 2012; Prasad et al., 2013).

Moreover, stimulant medication has been found effective to reduce susceptibility to auditory noise as well (Tillery et al., 2000; Freyaldenhoven et al., 2005). On the other hand, effects of medication on any of three central auditory processing measures are not found (Tillery et al., 2000). This may be regarded as good news for noise, as noise benefits can be seen in domains were medication has little or no effects, indicating that the working mechanisms of white noise and stimulant medication differ.

The working mechanisms of noise benefits are not yet known, but apart from SR, auditory masking is a good candidate in speech recognition, as a masker different from the signal, the noise can facilitate signal detection (Durlach et al., 2003). Furthermore, masking has been shown to have effect on impulsivity (Gray et al., 2002) but also in other modalities like in vision (Dawes et al., 2009), or the tactile sense (Tan et al., 2003). In both SR and masking tasks, irrelevant or meaningless stimulation in different modalities increases the signal-to-noise ratio and thus improves performance in various sensory or cognitive tasks. Yet another explanation to consider is that, instead of inducing SR, white noise increases arousal in participants. Such explanations are consistent with state regulation models of ADHD (Sonuga-Barke et al., 2010), derived from cognitive energetic theories (Sergeant, 2005). This theory posits that children with attention problems have difficulties in modulating their levels of arousal and activity in order to adjust to changing circumstances in the environment, particularly during boring tasks like speech recognition.

In future studies a sub-threshold noise condition should be added to determine the threshold speech signal. In this setting it is possible to investigate whether the relative benefit of noise for inattentive persons is apparent in threshold SR as well. Wong et al. (2008) found that in young adults, when listening to speech in low noise (20 dB below the speech signal), crucial networks in the auditory cortex and frontal areas were activated. One hypothesis, if speech processing deficits in ADHD are evident, could be that individuals with ADHD have dysfunctional neural pathways before the superior temporal gyrus and thus display difficulties in detecting signals at minimum or low audible levels. If this holds, external noise might induce increased network activation, involving more neuronal structures, thus producing higher level of internal noise in the brain, in line with predictions from the MBA model (Sikström and Söderlund, 2007).

### Limitations

This study should be regarded as a pilot insofar that no firm conclusions could be drawn from it because of the small number of participants; there were only 15 in the clinical, ADHD symptom group. Our findings have to be corroborated in a follow-up study. We do not know if these findings are valid for girls either since only boys participated.

Importantly, further studies should include testing in a sound proof setting rather in a school setting which involves a lot of ambient noise. Tone audiometry thresholds as well as speech recognition thresholds should be measured in the lab monaurally to further evaluate binaural speech recognition thresholds. Although all participants had hearing within normal range, the difference between the groups could be due to subtle differences in the peripheral transmission, e.g., in the middle ear or in the cochlea. However the correlation between hearing thresholds and the rating of ADHD symptoms speaks against this, implicating that the difference is due to a neurocognitive dimension rather than a perceptual peripheral deficit.

## Author Contributions

Shared first authorship, both authors have contributed to the outlining, the design, and planning of the study. Both have contributed significantly to the writing of the manuscript. ENJ had the main responsibility for data collection and test battery. GS has been responsible for the statistical assessment, figures, tables and the outlining of the discussion. Both authors have collaborated through the revision process and the final version of the discussion.

## Conflict of Interest Statement

The authors declare that the research was conducted in the absence of any commercial or financial relationships that could be construed as a potential conflict of interest.

The reviewer Staffan Hygge and the handling editor declare their shared affiliation, and the handling Editor states that the process nevertheless met the standards of a fair and objective review.
